# Safety and feasibility of a novel bi-directional portal vein access kit during transjugular intrahepatic portosystemic shunt creation

**DOI:** 10.1186/s42155-023-00366-x

**Published:** 2023-04-19

**Authors:** Richard D. Kang, Nariman Nezami, Peter Park, Anthony A. DePalma, Mohammed F. Loya, Rahul Mhaskar, Chad Engel, Bruce Zwiebel, Glenn Hoots, Jamil Shaikh

**Affiliations:** 1 University of South Florida, Morsani College of Medicine, Tampa, FL USA; 2 Division of Vascular and Interventional Radiology, Department of Diagnostic Radiology and Nuclear Medicine, University of Maryland School of Medicine, Baltimore, MD USA; 3 Experimental Therapeutics Program, University of Maryland Marlene and Stewart Greenebaum Comprehensive Cancer Center, Baltimore, MD USA; 4 Emory University, School of Medicine, Department of Radiology and Image Guided Medicine, Atlanta, GA USA; 5 Mid-Atlantic Permanente Medical Group, Kaiser Permanente, Rockville, MD USA; 6 Department of Radiology, University of South Florida Health, Tampa General Hospital, Tampa, FL USA

**Keywords:** Portal hypertension, TIPS, Bi-directional, Portal vein access

## Abstract

**Background:**

Transjugular intrahepatic portosystemic shunt (TIPS) creation remains as one of the more technically challenging endovascular procedures. Portal vein access from the hepatic vein often requires multiple needle passes, which increases procedure times, risk of complications, and radiation exposure. With its bi-directional maneuverability, the Scorpion X access kit may be a promising tool for easier portal vein access. However, the clinical safety and feasibility of this access kit has yet to be determined.

**Materials and methods:**

In this retrospective study, 17 patients (12 male, average age 56.6 ± 9.01) underwent TIPS procedure using Scorpion X portal vein access kits. The primary endpoint was time taken to access the portal vein from the hepatic vein. The most common indications for TIPS were refractory ascites (47.1%) and esophageal varices (17.6%). Radiation exposure, total number of needle passes, and intraoperative complications were recorded. Average MELD Score was 12.6 ± 3.39 (range: 8–20).

**Results:**

Portal vein cannulation was successfully achieved in 100% of patients during intracardiac echocardiography-assisted TIPS creation. Total fluoroscopy time was 39.31 ± 17.97 min; average radiation dose was 1036.76 ± 644.15 mGy, while average contrast dose was 120.59 ± 56.87 mL. The average number of passes from the hepatic vein to the portal vein was 2 (range: 1–6). Average time to access the portal vein once the TIPS cannula was positioned in the hepatic vein was 30.65 ± 18.64 min. There were no intraoperative complications.

**Conclusions:**

Clinical utilization of the Scorpion X bi-directional portal vein access kit is both safe and feasible. Utilizing this bi-directional access kit resulted in successful portal vein access with minimal intraoperative complications.

**Level of evidence:**

Retrospective cohort.

## Background

Transjugular intrahepatic portosystemic shunt (TIPS) creation is widely used for the management of sequelae of portal hypertension (Rösch et al. 1969, 1971; Vizzutti et al. 2020; Rajesh et al. 2020). While it was originally created for the treatment of esophageal varices, TIPS creation is now regularly performed to treat refractory ascites, hepatic hydrothorax, hepatorenal syndrome, portal hypertensive gastropathy and occasionally for portal vein thrombosis (Vizzutti et al. 2020; Rajesh et al. 2020; Tripathi et al. 2020). The procedure’s safety and widespread acceptance is largely attributable to significant technological advancements in the field of portal intervention, such as the development of partially covered expandable metallic stents and balloon angioplasty catheters (Keller et al. 2016). Despite these innovations, TIPS placement remains one of the more technically challenging endovascular procedures.

Traditionally, TIPS placement is performed under fluoroscopic guidance and wedged CO2 portography to identify and roadmap the portal venous anatomy. Needle passes are made from the selected hepatic vein into the target portal branch to obtain direct portohepatic venous access. The tract is subsequently dilated and stented. The most challenging step is the direct cannulation of the portal branch from the selected hepatic vein, as the needle pass is performed essentially blind in a three-dimensional volume with a two-dimensional roadmap for reference. To mitigate some of these guiding challenges, alternative image guidance options such as intracardiac echocardiography (ICE) catheter-guided portal access, wire-targeting access (gun-sight technique) and cone-beam computed tomography (CBCT)-guided access techniques have been introduced (Lukies et al. 2022; Trieu et al. 2017; Morrison et al. 2017; Lang et al. 2017; Shin et al. 2020).

**Fig. 1:**
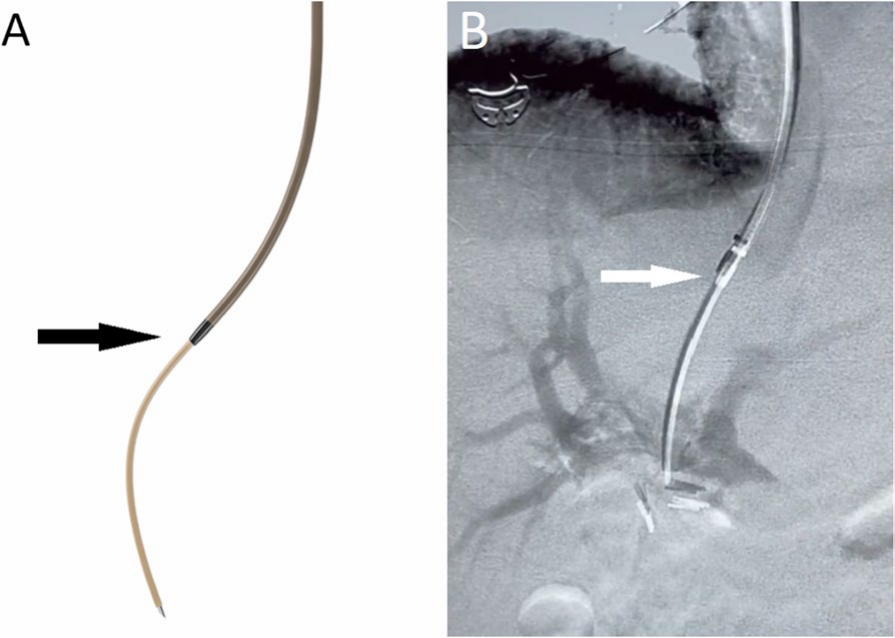
**A**. Schematic picture and **B**. Fluoroscopy image demonstrating access of the portal vein from the hepatic vein using the bi-directional functionality of the Scorpion X access kit. The inflection points between needle and cannula which highlight bi-directional movement are highlighted with arrows

Even with these new imaging techniques, multiple needle passes are often required for successful cannulation, especially for the inexperienced operator and in cases of more challenging portal venous anatomy. While innovations in TIPS creation technique have centered around improving image guidance for portal vein access, only minimal advancement was focused on improving the portal vein access devices. Recently, the Scorpion X (Argon Medical) bi-directional portal vein access kit (Fig. 1) was introduced to the market which allows for in-vivo needle access redirection through the parenchyma. The objective of this study is to evaluate the feasibility and safety of the Scorpion X portal vein access kit in obtaining portal vein access during TIPS creation.

## Materials and methods

### Study design and population

In this retrospective study, 17 patients with portal hypertension in need of portal decompression were studied from August 2021 to February 2022. Exclusion criteria included: hepatocellular carcinoma on pre procedural imaging, concurrent second malignancy, baseline end stage renal failure, chronic portal vein thrombosis or cavernous transformation of the portal vein on prior imaging examination which would impede access to the main portal vein, variant portal vein anatomy, post-sinusoidal portal hypertension (i.e. sinistral hypertension) or causes of portal hypertension other than end-stage liver disease (ESLD) and other concurrent disease processes that would limit the test subject in any capacity prior to the conclusion of the study.

Portal vein access was obtained by attending interventional radiologists with similar training (average experience 6 years) at tertiary level centers in the United States. All three centers are high volume liver transplant centers with the average number of TIPS procedures performed by the interventional radiology department at all three institutions averaging between 80–100 TIPS per year. All patients were evaluated by transplant team (surgeon and hepatologist) prior to the procedure for possible transplantation candidacy. Additionally, baseline contrast-enhanced multiphasic magnetic resonance imaging (MRI) or computed tomography (CT) of the abdomen was obtained prior to TIPS placement. Imaging was used to determine abnormal portal vein anatomy, concurrent underlying portal vein thrombosis and or baseline hepatocellular carcinoma. Careful attention was paid to delineate patient arterial and biliary structures to avoid complication. Imaging studies relevant to TIPS creation were interpreted by experienced abdominal radiologists.

Model for End-stage Liver Disease (MELD) scores were calculated for every patient prior to performing the procedure. Technical success was measured in time (minutes) required to access the portal vein once the TIPS cannula was positioned and directed in the hepatic vein prior to a pass. As a part of an effort to standardize TIPS procedures and reduce complications, incorporation of ICE ultrasound has become the standard operating procedure at our institutions. Assisted access using additional ancillary imaging modalities was noted, if necessary. Total radiation time, radiation dose, number of passes and total intraprocedural times were tracked. Immediate complications were recorded after the procedure including injury to adjacent structures or viscera. Postoperative complications were categorized as per Society of Interventional Radiology (SIR) adverse event classification. Shunt patency by duplex ultrasonography was documented at 1 month.

### Portal vein access technique

**Fig. 2:**
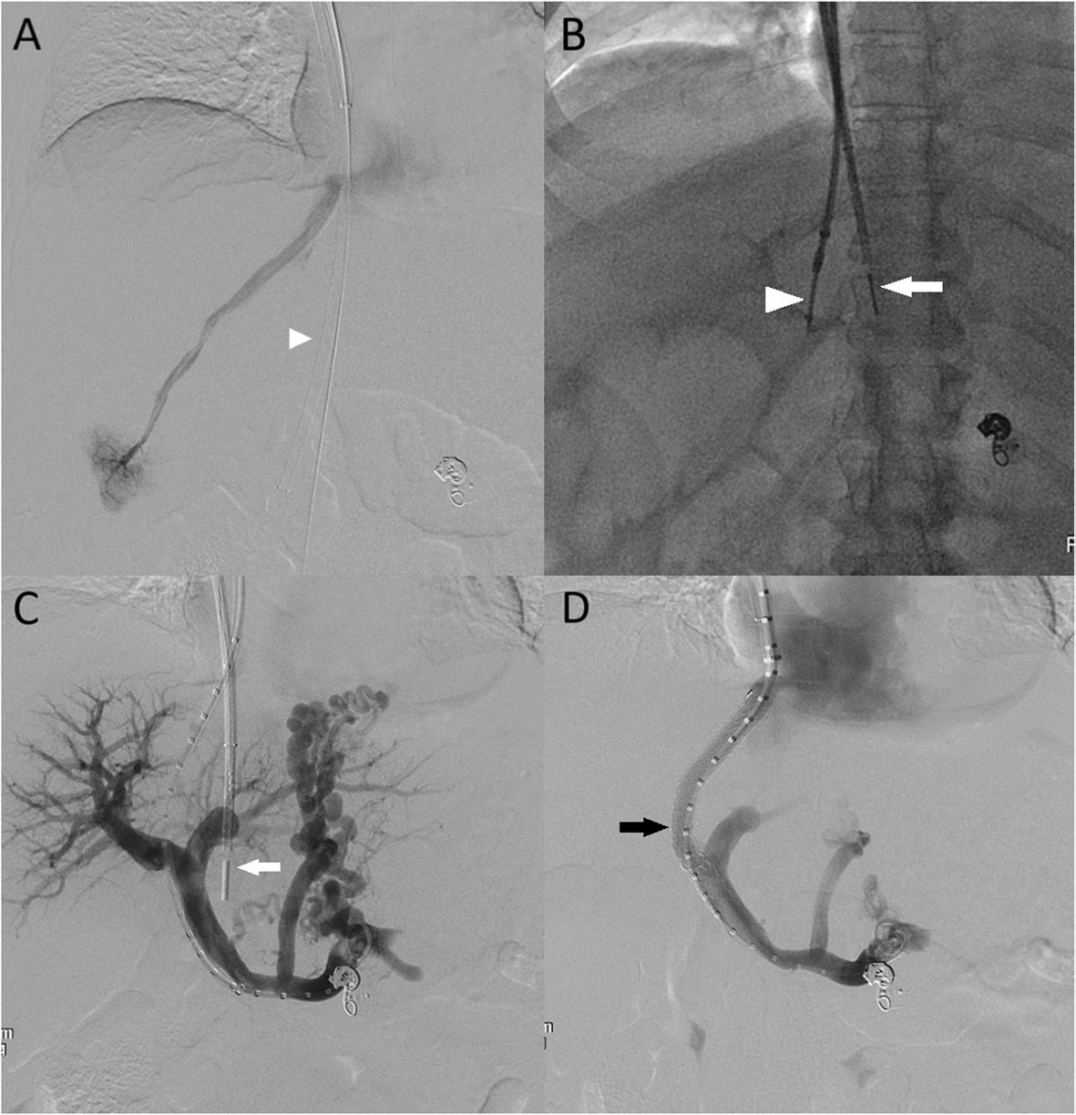
A Sample Case of Splenoportogram and TIPS placement. **A.** Right hepatic vein venogram with Blakemore tube noted in the background (white arrowhead). **B.** ICE catheter (white arrow) positioned at the level of the main portal vein. Advancement of the needle (white arrowhead) through hepatic vein towards main portal vein. **C.** Portovenogram through marked pigtail showing hepatopetal flow with multiple esophageal varices. The ICE catheter is positioned at the level of the right main portal vein (white arrow). **D.** Portovenogram demonstrating successful TIPS stent placement (black arrow) and significant reduction in opacification of the esophageal varices

**Fig. 3:**
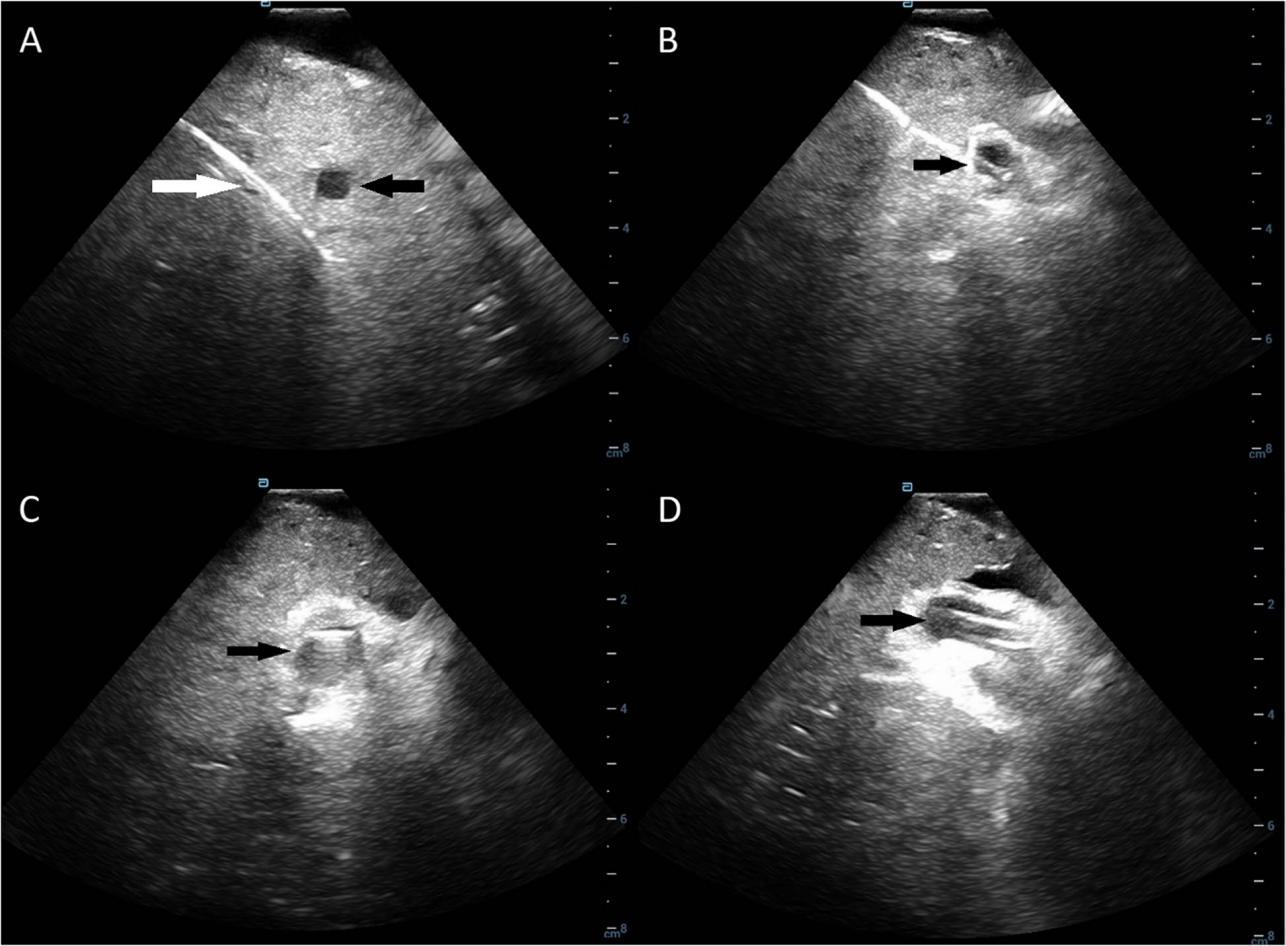
Portal Vein Access Under Intracardiac Echocardiography (ICE) Guidance. **A** US visualization of right hepatic vein catheterization (white arrow) with adjacent right portal vein (black arrow). **B** Advancement of the ICE catheter into the right portal vein (black arrow). **C** TIPS needle tip (black arrow) in the portal vein. **D** Advancement of the TIPS introducer sheath into the portal vein (black arrow)

All portal vein access creation during the TIPS procedure were performed by board-certified interventional radiologists with similar training (average experience 6 years). Greater than 90% of the procedures were performed by 1 of 3 interventionists, M.F.L., P.P., or J.S. Technical success was defined as access and passing of a wire into the main portal vein. All procedures were performed under general ansethesia per hospital protocol due to painful tract dilatation and technical complexity of the cases. Following standard micro puncture technique of the right internal jugular vein under ultrasound guidance, a 10F vascular sheath was advanced into the inferior vena cava (Flexor; Cook Medical, Bloomington, IN). A tandem access point was created in the right internal jugular vein for advancement of ICE catheter. In cases where tandem right internal jugular vein was inadequate, the right femoral vein was used instead. Depending on operator preference and institutional availability, one of two ICE catheters (EP Med ViewFlex Xtra; St. Jude Medical, Fullerton, CA) or (Acuson X300 AcuNav; Siemens Healthineers, Erlangen, Germany) was advanced to the level of the right portal vein (Fig. 2). Under ICE guidance, a 5F multipurpose angiographic catheter (Argon Medical, Athens, TX, USA) was advanced down the right internal jugular vein. Right atrial pressures were recorded and the catheter was advanced into the intended hepatic vein. A TIPS indroducer sheath was advanced over the catheter into the hepatic vein. The 13-G stiffening cannula of the Scorpion X (Argon Medicalm Athens, TX) access kit is advanced through the introducer sheath. A 6F polyether ether-ketone catheter containing a 17-gauge needle is placed into the safety funnel of the stiffening cannula. Using the ICE catheter as a landmark, the needle of the Scorpion X access kit was advanced incrementally until reaching the portal vein (Fig. 3). Access of the portal vein was confirmed using blood aspiration and contrast injection.

### Statistical analysis

Data was analyzed by statistical software (IBM SPSS statistics ver. 27, Chicago, IL, USA). The quantitative data are reported as mean ± standard deviation (range), while categorical data are reported as percentages.

## Results

### Demographic characteristics

The patients’ demographic characteristics are presented in Table 1*.* Of the 17 patients underwent TIPS placement, 12 were male and 5 were female. The mean age of the patients was 56.6 ± 9.01 years old, with BMI of 30.9 ± 6.58. The clinical indications of TIPS creation were ascites (47.1%) followed by esophageal varices (17.6%). The complete list of indications is outlined in Table 2. The mean MELD Score was 12.6 ± 3.4 (range: 8–20).

**Table 1:** Demographic Characteristics

Variables	Mean ± standard deviation
Age	56.6 ± 9.01
Sex (M/F)	12/5
BMI (kg/m^2^)	30.9 ± 6.58
Childs-Pugh Score	7.65 ± 1.58
MELD Score	12.6 ± 3.39

**Table 2:** Indications for TIPS Creation

Indications	Frequency (%)
Ascites	47.1
Esophageal Varices	17.6
Acute Portal Vein Thrombosis	5.9
Decrease Operative Risk Prior to Cholecystectomy	5.9
Duodenal Varices	5.9
Progression of Portal Vein Thrombosis on Anticoagulation	5.9

### Intraprocedural variables

Intraprocedural variables are listed in Table 3. The mean total fluoroscopy time was 39.3 ± 18.0 min. Average radiation dose was 1036.8 ± 644.2 mGy. Average contrast volume was 120.6 ± 56.9 mL. Average time to access the portal vein from the hepatic vein was 30.7 ± 18.6 min. The average number of passes was 2 (Range 1–6 passes between hepatic vein to portal vein). Two patients (11.8%) had mild bleeding from transcapsular passes identified by peritoneal drainage of ascitic fluid during the procedure. In all 17 cases, the TIPS cannula was not removed from the patients for readjustment. No major intraprocedural complications were encountered (defined as injury to adjacent structures or viscera, SIR Grade C-F).

**Table 3:** Intraoprocedural Variables

Variables	Mean ± standard deviation
Total Fluoroscopy time (min)	39.31 ± 17.97
Radiation Dose (mGy)	1036.76 ± 644.15
Contrast use (mL)	120.59 ± 56.87
Time to Access Portal Vein from Hepatic Vein (min)	30.65 ± 18.64
Average Number of Passes from Hepatic Vein to Portal Vein (n)	2 (Range: 1—6)
Intraoperative Complications (n)	0

### Postprocedural variables

Postprocedural variables are shown in Table 4. Three out of 17 patients (17.6%) developed hepatic encephalopathy postoperatively. No other minor (SIR grade A or B) postoperative complications were observed. Transient elevation in liver enzymes and bilirubin is noted in all patients, trended down within 2–5 days. TIPS placement after successful portal vein access was deferred in one patient with normal portosystemic pressure gradient (PSG). Of the remaining 16 patients, 10 had patent shunts 1 month after procedure, 2 had occluded shunts, and 3 were lost to follow up.

**Table 4:** Postprocedural Variables

Variables	Number (%)
Unexpected Complications (n)	0
Hepatic Encephalopathy (n)	3
Shunt Patency After 1 Month	10 patent, 2 occluded, 3 lost to follow-up

## Discussion

In our initial experience with utilizing the bidirectional Scorpion X access kit is safe and feasible, offering a promising alternative option to traditional kits which lack bi-directional maneuverability. We achieved 100% technical success rate across all attending interventional radiologists with low complication profiles in our initial experience using the bi-directional Scorpion X access kit. The use of the Scorpion X access kit allowed for equally low and acceptable levels of average fluoroscopy time, radiation dose and contrast volume when compared to published literature using standard TIPS access equipment (Gaba et al. 2011; Miller et al. 2003). No major intraoperative complications were observed (defined as SIR Grade C-F). The average number of needle passes required to reach the portal vein from the hepatic vein was 2 (range: 1–6). The average time to access the portal vein from the hepatic vein was 30.65 ± 18.64 min (time includes utilization of ICE guidance to align the needle with the portal vein).

TIPS creation has proven to be an essential procedure in the emergent and elective management of cirrhotic patients with portal hypertension. Despite its more frequent use and increasing indications, this remains a technically challenging procedure for most interventionalists primarily due to its oblique 3-dimensional course through the hepatic parenchyma from the target hepatic vein to the portal venous system. Further compounding the complexity of the procedure is operator experience, which can have a significant effect on procedure time, safety, radiation dose and overall success. In recent years, use of adjunctive imaging techniques such as ICE and wire-targeting technique have gained popularity and have greatly assisted in increasing success rates; however, portal vein access needles have remained largely unchanged.

Despite technological improvements multiple needle passes are often still required for successful cannulation of the portal system. To combat the need for multiple needle passes, one manufacturer has explored the idea of bi-directional portal vein access kits. In contrast to the traditional Rösch-Uchida or Ring kits, the Scorpion X access kit allows for flexible and independent movement of the cannula and needle allowing for easier readjustment when advancing through the liver. This improved maneuverability allows for in-vivo re-direction for desired trajectory to the portal system, which decreases the need for complete cannula removal, manual manipulation, and reinsertion.

The main perioperative complications of traditional fluoroscopic TIPS creation include inadvertent injuries to the liver capsule, extrahepatic portal vein, hepatic artery, biliary ducts as well as surrounding viscera (Gaba et al. 2011). The need for additional needle passes further increases these risks. Hepatic artery injury can occur in up to 6% of the cases, and clinically relevant biliary injury is reported in 5% of cases (Gaba et al. 2011; Miller et al. 2003; Hidajat et al. 2006). In addition, repeated cannulation prolongs procedure time (including on table anesthesia time) and increases radiation dose (Hidajat et al. 2006; Maleux et al. 2006). Lastly, CO2 extravasation is reported in the literature 1.8% of the time; this complication can lead to serious morbidities such as hepatic capsular laceration which is known to be a rare cause of immediate intraprocedural mortality (Keller et al. 2016; Maleux et al. 2006; Kew and Davies 2004). Hepatic encephalopathy is a common and well-documented postoperative complication of TIPS placement that occurs 25% to 50% of the time (Schindler et al. 2020). Our incidence of 17.6% is well within these reported estimates.

Limitations of our study include small sample size and retrospective study design. Intraoperative variables (number of needle passes, complications) were not compared with a control group using standard access kits available on the market currently (i.e., Rösch-Uchida or Ring kits). Future directions of this study should concentrate on comparative analysis of the Scorpion X portal vein access kits and other readily available TIPS creation devices on the market.

## Conclusions

Utilization of the Scorpion X access kits were safe and feasible, offering a promising alternative option to traditional kits which lack bi-directional maneuverability. Prospective studies are required to investigate the comparative efficacy of bidirectional portal venous access needles for TIPS creation.

## Data Availability

The datasets used and/or analysed during the current study are available from the corresponding author on reasonable request.

## Abbreviations

TIPS: Transjugular intrahepatic portosystemic shunt
ICE: Intracardiac echocardiography
CBCT: Cone-beam computed tomography
ESLD: End-stage liver disease
MRI: Magnetic resonance imaging
CT: Computed tomography
MELD: Model for End-stage Liver Disease
SIR: Society of Interventional Radiology
PSG: Portosystemic pressure gradient
